# Supplementary material for: RNA Viruses of Amblyomma variegatum and Rhipicephalus microplus and Cattle Susceptibility in the French Antilles
Source: Viruses. 2020 Jan 26;12(2):144. doi: 10.3390/v12020144 (PMC7077237; doi:10.3390/v12020144)
Supplement: Supplementary file 1 [file viruses-12-00144-s001.pdf]

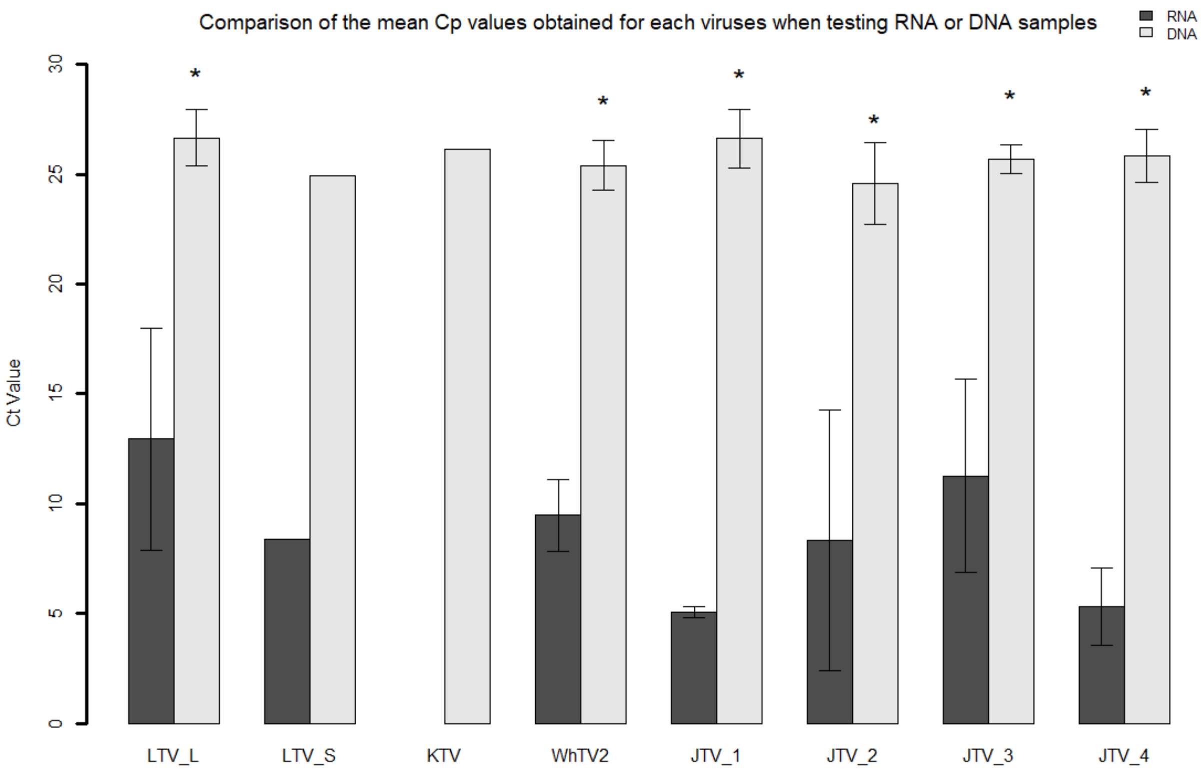

Figure S1. Research of endogeneous viral element (EVE) by virus screening in DNA samples: comparison of Cp values results obtained when detecting the viruses in DNA samples (Light gray) versus Cp values results obtained in the corresponding RNA samples (Dark gray). *: significative difference with *p*-value < 0.05 (T-test). The S segment of the LTV were found in only one DNA sample and in the corresponding RNA sample. KTV has been detected in one DNA sample but not in the corresponding RNA sample.

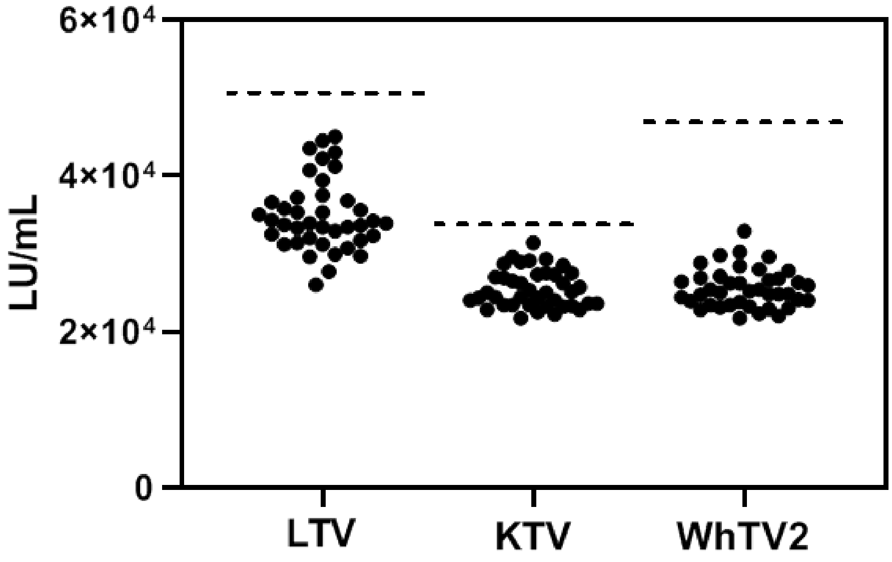

Figure S2. Luciferase activity (in LU/mL) distribution of measures after LIPS performed in tick/cattle interface for the screening of antibodies specific to Lihan tick virus (LTV), Karukera tick virus (KTV) and Wuhan tick virus 2 (WhTV2). Positivity threshold is indicated for each antigen construct with a dashed line.

Table S1. List of tick-borne viruses targeted by the microfluidic PCR system (Gondard *et al.,* 2018)

Family	Genus	Species
Asfarviridae	*Asfivirus*	African swine fever virus (ASFV)
Orthomyxoviridae	*Thogotovirus*	Thogoto virus (THOV)
		Dhori virus (DHOV)
Reoviridae	*Orbivirus*	Kemerovo virus (KEMV)
	Coltivirus	Colorado tick fever virus (CTFV)
		Eyach virus (EYAV)
Bunyaviridae	*Nairovirus*	Crimean-Congo Hemorrhagic fever virus (CCHF)
		Dugbe virus (DUGV)
		Nairobi sheep disease virus (NSDV)
	Phlebovirus	Uukuniemi virus (UUKV)
	Orthobunyavirus	Schmallenberg (SBV)
Flaviviridae	*Flavivirus*	Tick-borne encephalitis virus European subtype (TBE)
		Tick-borne encephalitis virus Far-Eastern subtype (TBE)
		Tick-borne encephalitis virus Siberian subtype (TBE)
		Louping ill virus (LIV)
		Langat virus (LGTV)
		Deer tick virus (DTV)
		Powassan virus (POWV)
		West Nile virus (WN)
		Meaban virus (MEAV)
		Omsk Hemorrhagic fever virus (OHFV)
		Kyasanur forest disease virus (KFDV)
